# The Engineering Side of Hospital Work

**Published:** 1905-06-10

**Authors:** 


### HOSPITAL ADMINISTRATION.

### CONSTRUCTION AND ECONOMICS.

### THE ENGINEERING SIDE OF HOSPITAL WORK.

XIV. ELECTRICAL APPARATUS.

(*Continued from page* 159.)

Electric Pressure.

Electric pressure is the electrical equivalent of mechanical pressure. Its unit, the volt, is used in the same way as the pound is used in mechanics, to indicate the ability to cause motion. In mechanics the motion may be of steam, air, water, or any substance, with electricity it is motion of electricity, or as we say an electric current, and the strength of the current passing between any two points, or between any two surfaces always depends directly upon the difference of pressure existing at any moment between the two points or surfaces. It is important to remember this law, as it appears to the writer to seriously affect the understanding of the action of electric currents upon, and in, the human body. Thus, when a difference of electrical pressure exists between, say, the two hands, the hands and the feet, or, as in the American method of electrocution, between the head and the feet, there is a continual and gradual lessening of the difference of pressure at different points in the body lying between the electrodes. Thus, where the hands are the electrodes the difference of pressure between the shoulders will be considerably less than between the hands. Where the hands and feet are the electrodes there will be considerably less difference of pressure between the shoulders and the hips than between the hands and the feet. And this may be followed right into the body, the difference of pressure becoming less and less as the distance, measured in electrical resistance, between the two points is lessened. But the point the writer wishes to bring out here is—at different points in the direct line between the two outside electrodes other paths will branch out, and the law mentioned above, that currents will pass through all of these paths, and in direct proportion to the difference of pressure existing at any moment between the ends of the respective paths, holds good, and is of considerable importance. Further, it appears to him that it should be possible to measure the difference of pressure existing at different points or surfaces on the outside of and within the body, and to approximately estimate the strength of the current that will pass between those points or surfaces when a given difference of pressure exists between two points on the surface of the body. The difference of electrical pressure between the two surfaces of a membrane may be of considerable importance, in view of its effect, as will be explained, on the osmotic pressure at the membrane. Before passing on another point had better be mentioned, as its importance is quite equal to that of the law enunciated above. The difference of pressure which governs the operation of the law is the net, or algebraical, sum of all the differences of pressure existing between the two points. The meaning of this is, there may be, and often are, more than one source of difference of pressure, and it is the algebraical sum of all of those that affect the particular paths in which electric currents are expected to pass, that must be used in the calculation. It may, and does happen, that the passage of an electric current creates an electric pressure more or less in opposition to the primary pressure, and this opposing pressure, or such proportion of it as is applicable to each path, must be taken into account when the calculation is made. The importance of this will be seen when it is mentioned, as will be explained, that to the presence of an opposing electrical pressure is due the fact that while, in New York, murderers are executed with pressures of 1,500 volts, or thereabouts, and several men have been killed accidentally with pressures of 200 volts, as in the case at the Fulham baths, and it is perfectly practicable to kill by electricity with less than 100 volts difference of pressure between the anode and cathode of the body, currents having pressures of 1,000,000 volts and over, are used with benefit in what is known as the high frequency treatment.

The Properties of Electric Currents. Electrical Resistance.

The strength of the electric current passing, upon which many of the phenomena of electricity depend, varies inversely as the resistance offered to its passage, and the resistance itself depends directly upon the length of the conductor through which the current is passing, and inversely upon its sectional area. It depends also upon the substance of which the conductor is composed, and upon the physical conditions present. The above must be taken in the widest sense, the resistance of any part of the body through which the current passes being subject to this law. It should also be noted that the passage of the current through a conductor frequently alters its resistance. Thus, in the case of the skin, the heat developed by the passage of the current at the electrodes increases the resistance offered by drying up the moisture present. The extreme case of this is where men have owed their lives to the fact that the arc formed between portions of their bodies, and a conductor with which they had come into contact, has cauterised the skin, and has so increased the resistance to the current that sufficient to do permanent injury could not pass. The ohm is the unit of resistance. It is the resistance offered by a certain column of mercury; that offered by other bodies is referred to thus, as a standard.

Generation of Heat.

Whenever a current of electricity passes through any substance, heat is generated, directly in proportion to the square of the pressure available at the points where the current enters, and where it leaves the substance, and inversely as the resistance offered by the substance. The formula is often given as the heat generated is proportional to the square of the current passing multiplied by the resistance. This formula is also true, but the first rule, it appears to the writer is the most useful, as it enables the surgeon, for instance, to judge the effect of adding cells to a battery used for heating a cautery. The pressure used in the calculation, it will be understood, must be that existing when the current is passing. This is sometimes lower than that measured when no current is passing, and is always so with batteries. The law applies equally to pressures arriving at points in the tissues, as to pressures on the surface of the skin. The number of heat units generated in any conductor by the passage of a current may be calculated by dividing either of the formulæ given above by 17·58, the pressures and resistances being in volts and ohms, and the rise in temperature may also be calculated, approximately, by bringing the specific heat of the substance into the equation.

Electrolysis.

Whenever a current of electricity passes through an electrolyte—nearly all known liquids are electrolytes—a portion of the liquid is decomposed, the non-metals produced by the decomposition turning to the anode, the point where the current enters the liquid, and the metals turning to the cathode. In this matter hydrogen behaves as a metal, always appearing at the negative pole. The quantity of the liquid decomposed depends directly upon the strength of the current passing, and upon the electro-chemical equivalent of the substances. The results of electrolysis often alter the resistances offered to the passage of the current, and introduce opposing pressures. Thus the generation of hydrogen at the negative pole always creates an opposing pressure, and an additional resistance. Electrolysis takes place wherever a current finds an electrolyte, and may take place in several successive electrolytes, if the current, or a part of it, passes through them. Thus, currents passing through the body will perform electrolysis upon the different liquids they meet, as they meet them.

Galvanic Action.

This is closely allied to electrolysis. Electrolysis always takes place in a galvanic battery, and the results of electrolysis produced by a current from any source may lead to galvanic action. Galvanic action will take place whenever there are two dissimilar substances present and a path for the current that is generated. Thus Galvani is credited with having made a galvanic battery from a number of heads of oxen, the skull forming one pole of each cell and the tongue the other. Tissues of different kinds, when in proximity with each other will create galvanic action, leading to small electric pressures, and small currents, if there are paths open for them. Instances of galvanic batteries, in general engineering, are copper wires held by iron staples, with moisture present, and the iron of steam boilers, which has a small electric pressure between parts that are at different temperatures.

*(To be continued.)*

